# What Caused Death

**Published:** 1894-08

**Authors:** J. D. Hopper


### What Caused Death.

I have a case I wish to report in the *Brief*, hoping to get some light on it.

Mrs. C., aged twenty-five years, married and the mother of four children. On May 14th and 15th she worked in her garden,. became over-heated, and after getting cool, began complaining of toothache. Jaw began to swell with sore throat. On Sunday following I was called to see her. Found her with all the symptoms of a severe case of tonsilitis. My treatment was in accordance with this diagnosis, but produced no effect. Fearing tracheotomy would have to be performed, consultation was called. Diagnosis was concurred in, but no operation was done. Patient seemed to improve a little as far as breathing was concerned, and would get so she could swallow a little every day. The swelling and pain increased, the latter being very severe, blood was drawn from the swelling to give relief which would only last a few minutes. On Thursday patient became a little delirious, for, a short while afterward, as I was passing her bed, she exclaimed: "Doctor, do you love Jesus?" and began shouting and praising God; called all her friends and children up to her and said: "I must die and leave you all." After quieting her as best I could, she began to sweat profusely, and the heart's action increased. This continued for twenty-four hours when death closed the scene. Was it blood poisoning, or what was it? She did not die from asphyxia.

I hope to have the opinion of the editor and some of the older practitioners. J. D. HOPPER, M.D.

